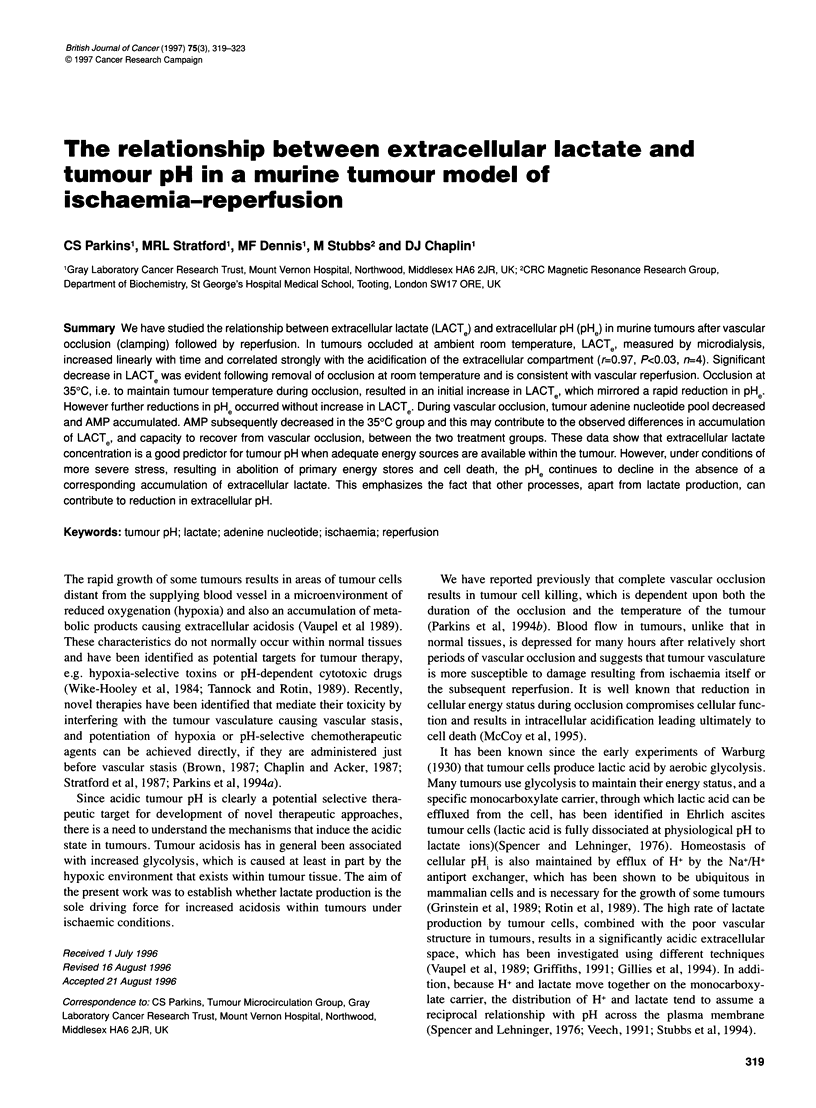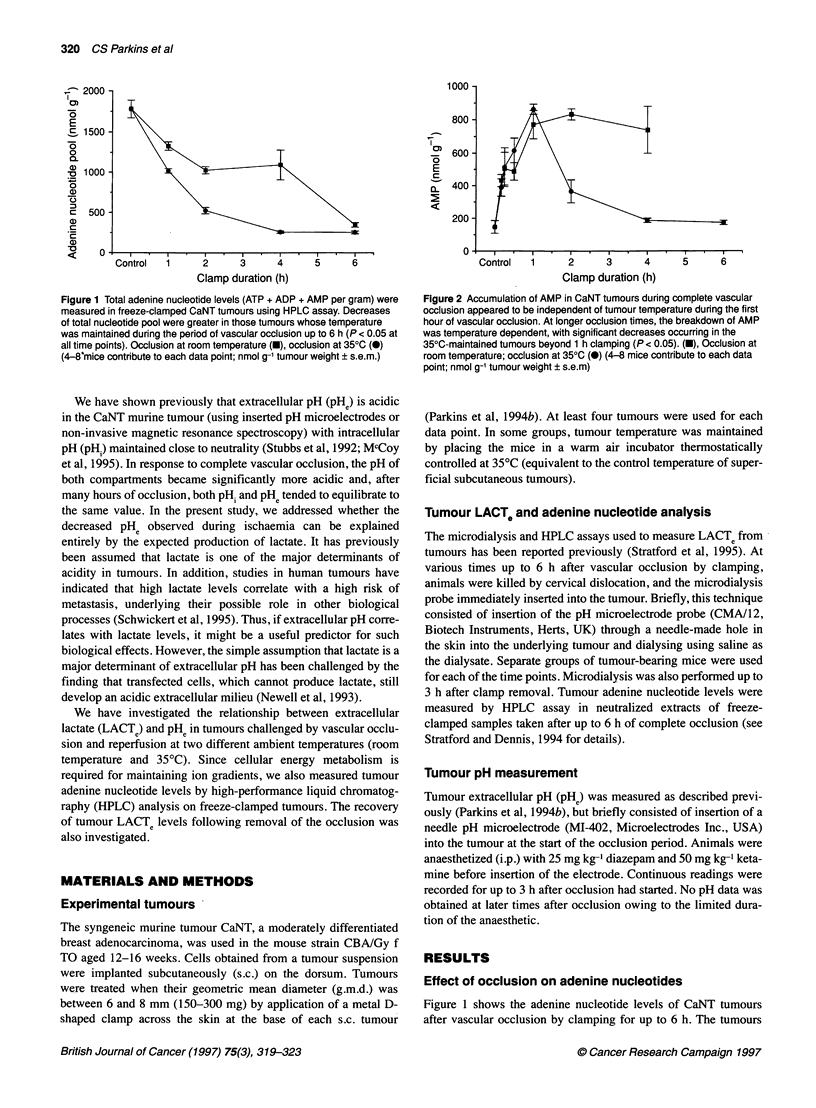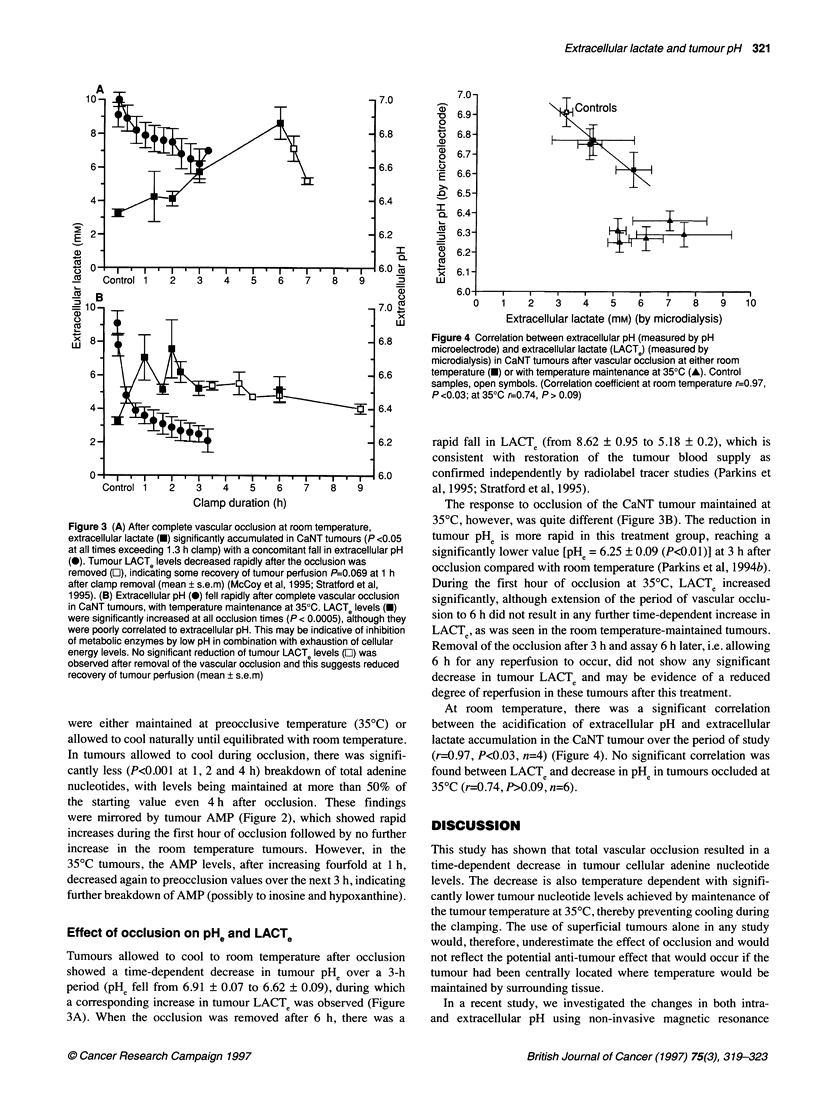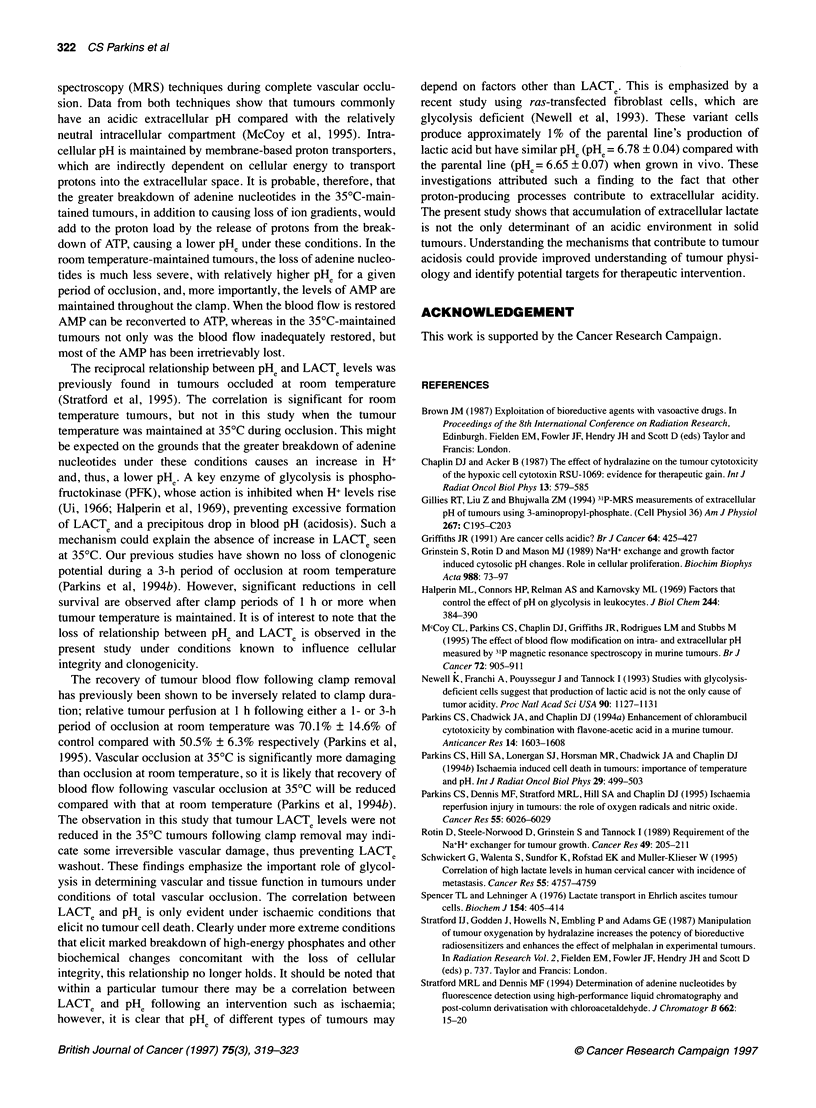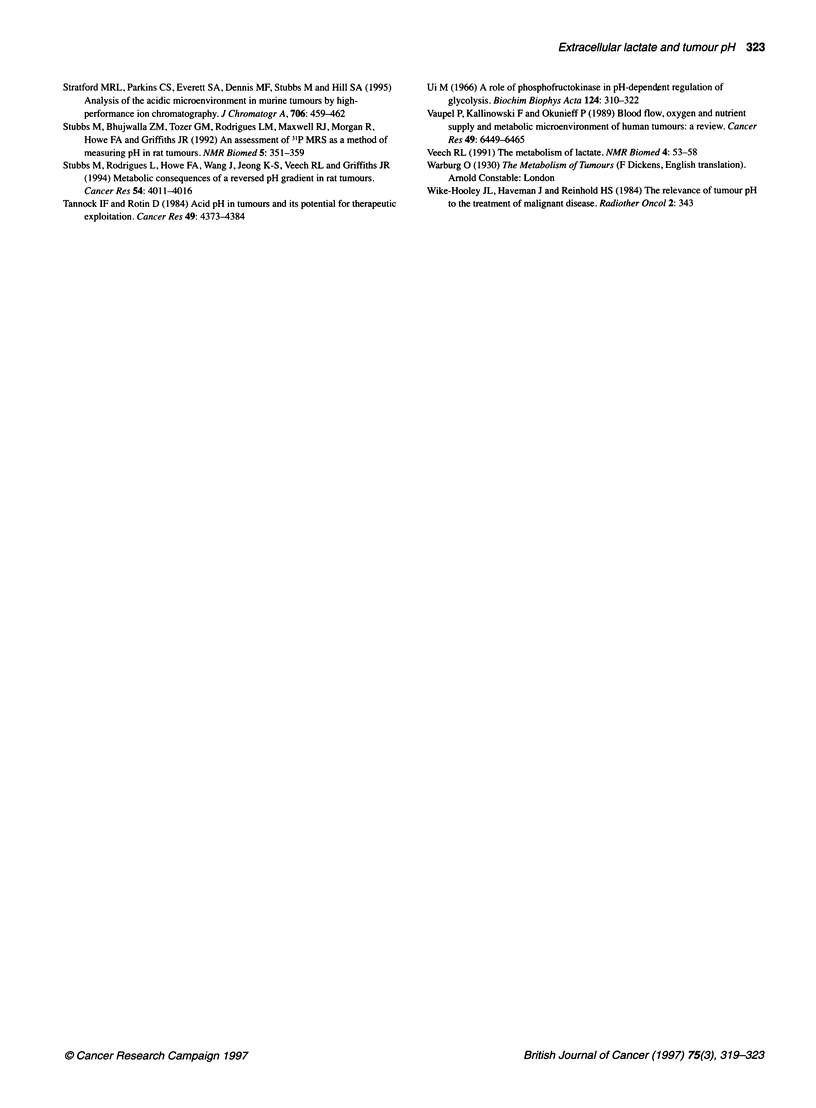# The relationship between extracellular lactate and tumour pH in a murine tumour model of ischaemia-reperfusion.

**DOI:** 10.1038/bjc.1997.53

**Published:** 1997

**Authors:** C. S. Parkins, M. R. Stratford, M. F. Dennis, M. Stubbs, D. J. Chaplin

**Affiliations:** Gray Laboratory Cancer Research Trust, Mount Vernon Hospital, Northwood, Middlesex, UK.

## Abstract

We have studied the relationship between extracellular lactate (LACTe) and extracellular pH (pHe) in murine tumours after vascular occlusion (clamping) followed by reperfusion. In tumours occluded at ambient room temperature, LACTe, measured by microdialysis, increased linearly with time and correlated strongly with the acidification of the extracellular compartment (r=0.97, P<0.03, n=4). Significant decrease in LACTe was evident following removal of occlusion at room temperature and is consistent with vascular reperfusion. Occlusion at 35 degrees C, i.e. to maintain tumour temperature during occlusion, resulted in an initial increase in LACTe, which mirrored a rapid reduction in pHe. However further reductions in pHe occurred without increase in LACTe. During vascular occlusion, tumour adenine nucleotide pool decreased and AMP accumulated. AMP subsequently decreased in the 35 degrees C group and this may contribute to the observed differences in accumulation of LACTe, and capacity to recover from vascular occlusion, between the two treatment groups. These data show that extracellular lactate concentration is a good predictor for tumour pH when adequate energy sources are available within the tumour. However, under conditions of more severe stress, resulting in abolition of primary energy stores and cell death, the pHe continues to decline in the absence of a corresponding accumulation of extracellular lactate. This emphasizes the fact that other processes, apart from lactate production, can contribute to reduction in extracellular pH.


British Journal of Cancer (1997) 75(3), 319–323
© 1997 Cancer Research Campaign

### The relationship between extracellular lactate and tumour pH in a murine tumour model of ischaemia–reperfusion

**CS Parkins[1], MRL Stratford[1], MF Dennis[1], M Stubbs[2] and DJ Chaplin[1]**

[1]Gray Laboratory Cancer Research Trust, Mount Vernon Hospital, Northwood, Middlesex HA6 2JR, UK; [2]CRC Magnetic Resonance Research Group, Department of Biochemistry, St George's Hospital Medical School, Tooting, London SW17 ORE, UK

**Summary** We have studied the relationship between extracellular lactate ($LACT_e$) and extracellular pH ($pH_e$) in murine tumours after vascular occlusion (clamping) followed by reperfusion. In tumours occluded at ambient room temperature, $LACT_e$, measured by microdialysis, increased linearly with time and correlated strongly with the acidification of the extracellular compartment ($r$=0.97, $P$<0.03, $n$=4). Significant decrease in $LACT_e$ was evident following removal of occlusion at room temperature and is consistent with vascular reperfusion. Occlusion at 35°C, i.e. to maintain tumour temperature during occlusion, resulted in an initial increase in $LACT_e$, which mirrored a rapid reduction in $pH_e$. However further reductions in $pH_e$ occurred without increase in $LACT_e$. During vascular occlusion, tumour adenine nucleotide pool decreased and AMP accumulated. AMP subsequently decreased in the 35°C group and this may contribute to the observed differences in accumulation of $LACT_e$, and capacity to recover from vascular occlusion, between the two treatment groups. These data show that extracellular lactate concentration is a good predictor for tumour pH when adequate energy sources are available within the tumour. However, under conditions of more severe stress, resulting in abolition of primary energy stores and cell death, the $pH_e$ continues to decline in the absence of a corresponding accumulation of extracellular lactate. This emphasizes the fact that other processes, apart from lactate production, can contribute to reduction in extracellular pH.

**Keywords:** tumour pH; lactate; adenine nucleotide; ischaemia; reperfusion

The rapid growth of some tumours results in areas of tumour cells distant from the supplying blood vessel in a microenvironment of reduced oxygenation (hypoxia) and also an accumulation of metabolic products causing extracellular acidosis (Vaupel et al 1989). These characteristics do not normally occur within normal tissues and have been identified as potential targets for tumour therapy, e.g. hypoxia-selective toxins or pH-dependent cytotoxic drugs (Wike-Hooley et al, 1984; Tannock and Rotin, 1989). Recently, novel therapies have been identified that mediate their toxicity by interfering with the tumour vasculature causing vascular stasis, and potentiation of hypoxia or pH-selective chemotherapeutic agents can be achieved directly, if they are administered just before vascular stasis (Brown, 1987; Chaplin and Acker, 1987; Stratford et al, 1987; Parkins et al, 1994*a*).

Since acidic tumour pH is clearly a potential selective therapeutic target for development of novel therapeutic approaches, there is a need to understand the mechanisms that induce the acidic state in tumours. Tumour acidosis has in general been associated with increased glycolysis, which is caused at least in part by the hypoxic environment that exists within tumour tissue. The aim of the present work was to establish whether lactate production is the sole driving force for increased acidosis within tumours under ischaemic conditions.

*Received 1 July 1996*
*Revised 16 August 1996*
*Accepted 21 August 1996*

*Correspondence to:* CS Parkins, Tumour Microcirculation Group, Gray Laboratory Cancer Research Trust, Mount Vernon Hospital, Northwood, Middlesex HA6 2JR, UK

We have reported previously that complete vascular occlusion results in tumour cell killing, which is dependent upon both the duration of the occlusion and the temperature of the tumour (Parkins et al, 1994*b*). Blood flow in tumours, unlike that in normal tissues, is depressed for many hours after relatively short periods of vascular occlusion and suggests that tumour vasculature is more susceptible to damage resulting from ischaemia itself or the subsequent reperfusion. It is well known that reduction in cellular energy status during occlusion compromises cellular function and results in intracellular acidification leading ultimately to cell death (McCoy et al, 1995).

It has been known since the early experiments of Warburg (1930) that tumour cells produce lactic acid by aerobic glycolysis. Many tumours use glycolysis to maintain their energy status, and a specific monocarboxylate carrier, through which lactic acid can be effluxed from the cell, has been identified in Ehrlich ascites tumour cells (lactic acid is fully dissociated at physiological pH to lactate ions)(Spencer and Lehninger, 1976). Homeostasis of cellular $pH_i$ is also maintained by efflux of $H^+$ by the $Na^+/H^+$ antiport exchanger, which has been shown to be ubiquitous in mammalian cells and is necessary for the growth of some tumours (Grinstein et al, 1989; Rotin et al, 1989). The high rate of lactate production by tumour cells, combined with the poor vascular structure in tumours, results in a significantly acidic extracellular space, which has been investigated using different techniques (Vaupel et al, 1989; Griffiths, 1991; Gillies et al, 1994). In addition, because $H^+$ and lactate move together on the monocarboxylate carrier, the distribution of $H^+$ and lactate tend to assume a reciprocal relationship with pH across the plasma membrane (Spencer and Lehninger, 1976; Veech, 1991; Stubbs et al, 1994).

**Figure 1** Total adenine nucleotide levels (ATP + ADP + AMP per gram) were measured in freeze-clamped CaNT tumours using HPLC assay. Decreases of total nucleotide pool were greater in those tumours whose temperature was maintained during the period of vascular occlusion up to 6 h ($P < 0.05$ at all time points). Occlusion at room temperature (■), occlusion at 35°C (●) (4–8 mice contribute to each data point; nmol $g^{-1}$ tumour weight ± s.e.m.)

**Figure 2** Accumulation of AMP in CaNT tumours during complete vascular occlusion appeared to be independent of tumour temperature during the first hour of vascular occlusion. At longer occlusion times, the breakdown of AMP was temperature dependent, with significant decreases occurring in the 35°C-maintained tumours beyond 1 h clamping ($P < 0.05$). (■), Occlusion at room temperature; occlusion at 35°C (●) (4–8 mice contribute to each data point; nmol $g^{-1}$ tumour weight ± s.e.m)

We have shown previously that extracellular pH ($pH_e$) is acidic in the CaNT murine tumour (using inserted pH microelectrodes or non-invasive magnetic resonance spectroscopy) with intracellular pH ($pH_i$) maintained close to neutrality (Stubbs et al, 1992; McCoy et al, 1995). In response to complete vascular occlusion, the pH of both compartments became significantly more acidic and, after many hours of occlusion, both $pH_i$ and $pH_e$ tended to equilibrate to the same value. In the present study, we addressed whether the decreased $pH_e$ observed during ischaemia can be explained entirely by the expected production of lactate. It has previously been assumed that lactate is one of the major determinants of acidity in tumours. In addition, studies in human tumours have indicated that high lactate levels correlate with a high risk of metastasis, underlying their possible role in other biological processes (Schwickert et al, 1995). Thus, if extracellular pH correlates with lactate levels, it might be a useful predictor for such biological effects. However, the simple assumption that lactate is a major determinant of extracellular pH has been challenged by the finding that transfected cells, which cannot produce lactate, still develop an acidic extracellular milieu (Newell et al, 1993).

We have investigated the relationship between extracellular lactate ($LACT_e$) and $pH_e$ in tumours challenged by vascular occlusion and reperfusion at two different ambient temperatures (room temperature and 35°C). Since cellular energy metabolism is required for maintaining ion gradients, we also measured tumour adenine nucleotide levels by high-performance liquid chromatography (HPLC) analysis on freeze-clamped tumours. The recovery of tumour $LACT_e$ levels following removal of the occlusion was also investigated.

### MATERIALS AND METHODS

### Experimental tumours

The syngeneic murine tumour CaNT, a moderately differentiated breast adenocarcinoma, was used in the mouse strain CBA/Gy f TO aged 12–16 weeks. Cells obtained from a tumour suspension were implanted subcutaneously (s.c.) on the dorsum. Tumours were treated when their geometric mean diameter (g.m.d.) was between 6 and 8 mm (150–300 mg) by application of a metal D-shaped clamp across the skin at the base of each s.c. tumour (Parkins et al, 1994*b*). At least four tumours were used for each data point. In some groups, tumour temperature was maintained by placing the mice in a warm air incubator thermostatically controlled at 35°C (equivalent to the control temperature of superficial subcutaneous tumours).

### Tumour $LACT_e$ and adenine nucleotide analysis

The microdialysis and HPLC assays used to measure $LACT_e$ from tumours has been reported previously (Stratford et al, 1995). At various times up to 6 h after vascular occlusion by clamping, animals were killed by cervical dislocation, and the microdialysis probe immediately inserted into the tumour. Briefly, this technique consisted of insertion of the pH microelectrode probe (CMA/12, Biotech Instruments, Herts, UK) through a needle-made hole in the skin into the underlying tumour and dialysing using saline as the dialysate. Separate groups of tumour-bearing mice were used for each of the time points. Microdialysis was also performed up to 3 h after clamp removal. Tumour adenine nucleotide levels were measured by HPLC assay in neutralized extracts of freeze-clamped samples taken after up to 6 h of complete occlusion (see Stratford and Dennis, 1994 for details).

### Tumour pH measurement

Tumour extracellular pH ($pH_e$) was measured as described previously (Parkins et al, 1994*b*), but briefly consisted of insertion of a needle pH microelectrode (MI-402, Microelectrodes Inc., USA) into the tumour at the start of the occlusion period. Animals were anaesthetized (i.p.) with 25 mg $kg^{-1}$ diazepam and 50 mg $kg^{-1}$ ketamine before insertion of the electrode. Continuous readings were recorded for up to 3 h after occlusion had started. No pH data was obtained at later times after occlusion owing to the limited duration of the anaesthetic.

### RESULTS

### Effect of occlusion on adenine nucleotides

Figure 1 shows the adenine nucleotide levels of CaNT tumours after vascular occlusion by clamping for up to 6 h. The tumours

© Cancer Research Campaign 1997

**Figure 3** (**A**) After complete vascular occlusion at room temperature, extracellular lactate (■) significantly accumulated in CaNT tumours ($P$<0.05 at all times exceeding 1.3 h clamp) with a concomitant fall in extracellular pH (●). Tumour $LACT_e$ levels decreased rapidly after the occlusion was removed (□), indicating some recovery of tumour perfusion $P$=0.069 at 1 h after clamp removal (mean ± s.e.m) (McCoy et al, 1995; Stratford et al, 1995). (**B**) Extracellular pH (●) fell rapidly after complete vascular occlusion in CaNT tumours, with temperature maintenance at 35°C. $LACT_e$ levels (■) were significantly increased at all occlusion times ($P$ < 0.0005), although they were poorly correlated to extracellular pH. This may be indicative of inhibition of metabolic enzymes by low pH in combination with exhaustion of cellular energy levels. No significant reduction of tumour $LACT_e$ levels (□) was observed after removal of the vascular occlusion and this suggests reduced recovery of tumour perfusion (mean ± s.e.m)

**Figure 4** Correlation between extracellular pH (measured by pH microelectrode) and extracellular lactate ($LACT_e$) (measured by microdialysis) in CaNT tumours after vascular occlusion at either room temperature (■) or with temperature maintenance at 35°C (▲). Control samples, open symbols. (Correlation coefficient at room temperature $r$=0.97, $P$ <0.03; at 35°C $r$=0.74, $P$ > 0.09)

were either maintained at preocclusive temperature (35°C) or allowed to cool naturally until equilibrated with room temperature. In tumours allowed to cool during occlusion, there was significantly less ($P$<0.001 at 1, 2 and 4 h) breakdown of total adenine nucleotides, with levels being maintained at more than 50% of the starting value even 4 h after occlusion. These findings were mirrored by tumour AMP (Figure 2), which showed rapid increases during the first hour of occlusion followed by no further increase in the room temperature tumours. However, in the 35°C tumours, the AMP levels, after increasing fourfold at 1 h, decreased again to preocclusion values over the next 3 h, indicating further breakdown of AMP (possibly to inosine and hypoxanthine).

### Effect of occlusion on $pH_e$ and $LACT_e$

Tumours allowed to cool to room temperature after occlusion showed a time-dependent decrease in tumour $pH_e$ over a 3-h period ($pH_e$ fell from 6.91 ± 0.07 to 6.62 ± 0.09), during which a corresponding increase in tumour $LACT_e$ was observed (Figure 3A). When the occlusion was removed after 6 h, there was a rapid fall in $LACT_e$ (from 8.62 ± 0.95 to 5.18 ± 0.2), which is consistent with restoration of the tumour blood supply as confirmed independently by radiolabel tracer studies (Parkins et al, 1995; Stratford et al, 1995).

The response to occlusion of the CaNT tumour maintained at 35°C, however, was quite different (Figure 3B). The reduction in tumour $pH_e$ is more rapid in this treatment group, reaching a significantly lower value [$pH_e$ = 6.25 ± 0.09 ($P$<0.01)] at 3 h after occlusion compared with room temperature (Parkins et al, 1994*b*). During the first hour of occlusion at 35°C, $LACT_e$ increased significantly, although extension of the period of vascular occlusion to 6 h did not result in any further time-dependent increase in $LACT_e$, as was seen in the room temperature-maintained tumours. Removal of the occlusion after 3 h and assay 6 h later, i.e. allowing 6 h for any reperfusion to occur, did not show any significant decrease in tumour $LACT_e$ and may be evidence of a reduced degree of reperfusion in these tumours after this treatment.

At room temperature, there was a significant correlation between the acidification of extracellular pH and extracellular lactate accumulation in the CaNT tumour over the period of study ($r$=0.97, $P$<0.03, $n$=4) (Figure 4). No significant correlation was found between $LACT_e$ and decrease in $pH_e$ in tumours occluded at 35°C ($r$=0.74, $P$>0.09, $n$=6).

### DISCUSSION

This study has shown that total vascular occlusion resulted in a time-dependent decrease in tumour cellular adenine nucleotide levels. The decrease is also temperature dependent with significantly lower tumour nucleotide levels achieved by maintenance of the tumour temperature at 35°C, thereby preventing cooling during the clamping. The use of superficial tumours alone in any study would, therefore, underestimate the effect of occlusion and would not reflect the potential anti-tumour effect that would occur if the tumour had been centrally located where temperature would be maintained by surrounding tissue.

In a recent study, we investigated the changes in both intra- and extracellular pH using non-invasive magnetic resonance

© Cancer Research Campaign 1997

spectroscopy (MRS) techniques during complete vascular occlusion. Data from both techniques show that tumours commonly have an acidic extracellular pH compared with the relatively neutral intracellular compartment (McCoy et al, 1995). Intracellular pH is maintained by membrane-based proton transporters, which are indirectly dependent on cellular energy to transport protons into the extracellular space. It is probable, therefore, that the greater breakdown of adenine nucleotides in the 35°C-maintained tumours, in addition to causing loss of ion gradients, would add to the proton load by the release of protons from the breakdown of ATP, causing a lower $pH_e$ under these conditions. In the room temperature-maintained tumours, the loss of adenine nucleotides is much less severe, with relatively higher $pH_e$ for a given period of occlusion, and, more importantly, the levels of AMP are maintained throughout the clamp. When the blood flow is restored AMP can be reconverted to ATP, whereas in the 35°C-maintained tumours not only was the blood flow inadequately restored, but most of the AMP has been irretrievably lost.

The reciprocal relationship between $pH_e$ and $LACT_e$ levels was previously found in tumours occluded at room temperature (Stratford et al, 1995). The correlation is significant for room temperature tumours, but not in this study when the tumour temperature was maintained at 35°C during occlusion. This might be expected on the grounds that the greater breakdown of adenine nucleotides under these conditions causes an increase in $H^+$ and, thus, a lower $pH_e$. A key enzyme of glycolysis is phosphofructokinase (PFK), whose action is inhibited when $H^+$ levels rise (Ui, 1966; Halperin et al, 1969), preventing excessive formation of $LACT_e$ and a precipitous drop in blood pH (acidosis). Such a mechanism could explain the absence of increase in $LACT_e$ seen at 35°C. Our previous studies have shown no loss of clonogenic potential during a 3-h period of occlusion at room temperature (Parkins et al, 1994*b*). However, significant reductions in cell survival are observed after clamp periods of 1 h or more when tumour temperature is maintained. It is of interest to note that the loss of relationship between $pH_e$ and $LACT_e$ is observed in the present study under conditions known to influence cellular integrity and clonogenicity.

The recovery of tumour blood flow following clamp removal has previously been shown to be inversely related to clamp duration; relative tumour perfusion at 1 h following either a 1- or 3-h period of occlusion at room temperature was 70.1% ± 14.6% of control compared with 50.5% ± 6.3% respectively (Parkins et al, 1995). Vascular occlusion at 35°C is significantly more damaging than occlusion at room temperature, so it is likely that recovery of blood flow following vascular occlusion at 35°C will be reduced compared with that at room temperature (Parkins et al, 1994*b*). The observation in this study that tumour $LACT_e$ levels were not reduced in the 35°C tumours following clamp removal may indicate some irreversible vascular damage, thus preventing $LACT_e$ washout. These findings emphasize the important role of glycolysis in determining vascular and tissue function in tumours under conditions of total vascular occlusion. The correlation between $LACT_e$ and $pH_e$ is only evident under ischaemic conditions that elicit no tumour cell death. Clearly under more extreme conditions that elicit marked breakdown of high-energy phosphates and other biochemical changes concomitant with the loss of cellular integrity, this relationship no longer holds. It should be noted that within a particular tumour there may be a correlation between $LACT_e$ and $pH_e$ following an intervention such as ischaemia; however, it is clear that $pH_e$ of different types of tumours may depend on factors other than $LACT_e$. This is emphasized by a recent study using *ras*-transfected fibroblast cells, which are glycolysis deficient (Newell et al, 1993). These variant cells produce approximately 1% of the parental line's production of lactic acid but have similar $pH_e$ ($pH_e = 6.78 \pm 0.04$) compared with the parental line ($pH_e = 6.65 \pm 0.07$) when grown in vivo. These investigations attributed such a finding to the fact that other proton-producing processes contribute to extracellular acidity. The present study shows that accumulation of extracellular lactate is not the only determinant of an acidic environment in solid tumours. Understanding the mechanisms that contribute to tumour acidosis could provide improved understanding of tumour physiology and identify potential targets for therapeutic intervention.

### ACKNOWLEDGEMENT

This work is supported by the Cancer Research Campaign.

### REFERENCES

Brown JM (1987) Exploitation of bioreductive agents with vasoactive drugs. In *Proceedings of the 8th International Conference on Radiation Research*, Edinburgh. Fielden EM, Fowler JF, Hendry JH and Scott D (eds) Taylor and Francis: London.

Chaplin DJ and Acker B (1987) The effect of hydralazine on the tumour cytotoxicity of the hypoxic cell cytotoxin RSU-1069: evidence for therapeutic gain. *Int J Radiat Oncol Biol Phys* **13**: 579–585

Gillies RT, Liu Z and Bhujwalla ZM (1994) $^{31}$P-MRS measurements of extracellular pH of tumours using 3-aminopropyl-phosphate. (Cell Physiol 36) *Am J Physiol* **267**: C195–C203

Griffiths JR (1991) Are cancer cells acidic? *Br J Cancer* **64**: 425–427

Grinstein S, Rotin D and Mason MJ (1989) $Na^+H^+$ exchange and growth factor induced cytosolic pH changes. Role in cellular proliferation. *Biochim Biophys Acta* **988**: 73–97

Halperin ML, Connors HP, Relman AS and Karnovsky ML (1969) Factors that control the effect of pH on glycolysis in leukocytes. *J Biol Chem* **244**: 384–390

McCoy CL, Parkins CS, Chaplin DJ, Griffiths JR, Rodrigues LM and Stubbs M (1995) The effect of blood flow modification on intra- and extracellular pH measured by $^{31}$P magnetic resonance spectroscopy in murine tumours. *Br J Cancer* **72**: 905–911

Newell K, Franchi A, Pouyssegur J and Tannock I (1993) Studies with glycolysis-deficient cells suggest that production of lactic acid is not the only cause of tumor acidity. *Proc Natl Acad Sci USA* **90**: 1127–1131

Parkins CS, Chadwick JA, and Chaplin DJ (1994*a*) Enhancement of chlorambucil cytotoxicity by combination with flavone-acetic acid in a murine tumour. *Anticancer Res* **14**: 1603–1608

Parkins CS, Hill SA, Lonergan SJ, Horsman MR, Chadwick JA and Chaplin DJ (1994*b*) Ischaemia induced cell death in tumours: importance of temperature and pH. *Int J Radiat Oncol Biol Phys* **29**: 499–503

Parkins CS, Dennis MF, Stratford MRL, Hill SA and Chaplin DJ (1995) Ischaemia reperfusion injury in tumours: the role of oxygen radicals and nitric oxide. *Cancer Res* **55**: 6026–6029

Rotin D, Steele-Norwood D, Grinstein S and Tannock I (1989) Requirement of the $Na^+H^+$ exchanger for tumour growth. *Cancer Res* **49**: 205–211

Schwickert G, Walenta S, Sundfor K, Rofstad EK and Muller-Klieser W (1995) Correlation of high lactate levels in human cervical cancer with incidence of metastasis. *Cancer Res* **55**: 4757–4759

Spencer TL and Lehninger A (1976) Lactate transport in Ehrlich ascites tumour cells. *Biochem J* **154**: 405–414

Stratford IJ, Godden J, Howells N, Embling P and Adams GE (1987) Manipulation of tumour oxygenation by hydralazine increases the potency of bioreductive radiosensitizers and enhances the effect of melphalan in experimental tumours. In *Radiation Research Vol. 2*, Fielden EM, Fowler JF, Hendry JH and Scott D (eds) p. 737. Taylor and Francis: London.

Stratford MRL and Dennis MF (1994) Determination of adenine nucleotides by fluorescence detection using high-performance liquid chromatography and post-column derivatisation with chloroacetaldehyde. *J Chromatogr B* **662**: 15–20

© Cancer Research Campaign 1997

Stratford MRL, Parkins CS, Everett SA, Dennis MF, Stubbs M and Hill SA (1995) Analysis of the acidic microenvironment in murine tumours by high-performance ion chromatography. *J Chromatogr A*, **706**: 459–462

Stubbs M, Bhujwalla ZM, Tozer GM, Rodrigues LM, Maxwell RJ, Morgan R, Howe FA and Griffiths JR (1992) An assessment of $^{31}$P MRS as a method of measuring pH in rat tumours. *NMR Biomed* **5**: 351–359

Stubbs M, Rodrigues L, Howe FA, Wang J, Jeong K-S, Veech RL and Griffiths JR (1994) Metabolic consequences of a reversed pH gradient in rat tumours. *Cancer Res* **54**: 4011–4016

Tannock IF and Rotin D (1984) Acid pH in tumours and its potential for therapeutic exploitation. *Cancer Res* **49**: 4373–4384

Ui M (1966) A role of phosphofructokinase in pH-dependent regulation of glycolysis. *Biochim Biophys Acta* **124**: 310–322

Vaupel P, Kallinowski F and Okunieff P (1989) Blood flow, oxygen and nutrient supply and metabolic microenvironment of human tumours: a review. *Cancer Res* **49**: 6449–6465

Veech RL (1991) The metabolism of lactate. *NMR Biomed* **4**: 53–58

Warburg O (1930) *The Metabolism of Tumours* (F Dickens, English translation). Arnold Constable: London

Wike-Hooley JL, Haveman J and Reinhold HS (1984) The relevance of tumour pH to the treatment of malignant disease. *Radiother Oncol* **2**: 343

© Cancer Research Campaign 1997